# Predictors of Stature Concerns among Young Chinese Women and Men

**DOI:** 10.3389/fpsyg.2017.01248

**Published:** 2017-07-20

**Authors:** Qingqing Sun

**Affiliations:** College of Ideological and Political Education, Henan University of Economics and Law Zhengzhou, China

**Keywords:** stature concerns, sociocultural pressure, objectification theory, stature surveillance, Chinese, youth

## Abstract

Stature concerns are a prominent source of body dissatisfaction for Chinese teenagers and young adults, yet little is known about the psychological factors that account for it. Therefore, this study examined social cultural model and objectification theory as explanations for stature concerns in a sample of undergraduate men and women from a university in Henan, China. Given height is a salient physical attribute for Chinese adolescents and young adults, we extended past studies on objectification theory by adding separate measures for stature surveillance. Participants (231 men, 473 women) completed a questionnaire assaying measures of sociocultural model features (appearance pressure from mass media and close interpersonal networks, appearance social comparisons), objectified body consciousness (body surveillance, body shame, stature surveillance), and stature concerns. In multiple regression models for each gender, appearance pressure from the mass media and stature surveillance were robust predictors of stature concerns for both genders, independent of reported height. Body surveillance predicted stature concerns for women but not men. These findings contribute to the broader field of multicultural body image research and may help to account for specific culturally salient appearance concerns within samples of young Chinese women and men.

## Introduction

A considerable body of research literature suggests that body dissatisfaction is a common concern for adolescents and young adults from Western countries (Ricciardelli and McCabe, 2001; Bearman et al., 2006; Neighbors et al., 2008). Like many Western kids, Chinese adolescents and young adults also expressed dissatisfaction with body size and shape (e.g., Chen et al., 2006, 2007; Chen and Jackson, 2008). However, in contrast to people in the West who focus exclusively on dissatisfaction with body size/weight (e.g., Ricciardelli and McCabe, 2001; Neighbors et al., 2008), Chinese adolescent and young adult males and females reported significantly more concern about being short than about being fat and facial appearance (Chen et al., 2006). This shows that concern with being short is one of the most common appearance concerns of Chinese adolescents and young adults. Some scholars posit that dissatisfaction with physical stature is a peculiar manifestation of Chinese negative physical self (Chen et al., 2006), and as such is a specific, culturally salient source of appearance dissatisfaction (Chen et al., 2006; Jackson and Chen, 2015b).

Research has demonstrated that stature concerns could cause some negative psychological consequences. For example, Chen et al. (2006) found that stature concerns were negatively associated with appearance self-esteem, physical sense of self-worth, and general appearance satisfaction in a large sample of Chinese adolescents and young adults. In addition, some individuals who are dissatisfied with their height may even turn to expensive, high-risk, and exceedingly painful leg lengthening surgery (Watts, 2004; Campens et al., 2010). Therefore, it is important and necessary to identify the psychological factors that account for stature concerns among young women and men in China.

To date, there has been a lack of research examining the predictors of these stature concerns. Numerous studies have demonstrated that sociocultural influences play a significant role in promoting body dissatisfaction (e.g., Keery et al., 2004; Smolak et al., 2005; Shroff and Thompson, 2006). Specifically, appearance pressure from the mass media, friends, and family deliver unrealistic societal standards of physical beauty. Because such body ideals cannot be attained by most, appearance pressure contributes to body dissatisfaction both directly and indirectly through increasing preferences of unrealistic attractiveness standards and the frequency of appearance comparisons with peers and media ideal (e.g., Thompson et al., 1999; Jones, 2004). Several studies have shown that sociocultural factors can also explain body dissatisfaction among young males and females in China. For example, Chen et al. (2007) found that teasing and social pressure to be thin directly predicted body dissatisfaction in a large sample of Chinese adolescents and young adults. Recently, Jackson et al. (2015) found that appearance pressure from the media predicted body dissatisfaction of Chinese female and male college students. However, that study focused largely on weight concerns; fewer studies have explored other specific body image problems (Jackson and Chen, 2008b).

Some evidence suggests that sociocultural factors may also play a significant role in promoting stature dissatisfaction. Reports have shown that in China, attractiveness ideals depicted by the media promote tall stature (Dai, 2007, unpublished; Hua, 2013, unpublished). For example, Hua (2013, unpublished) analyzed the Chinese edition of “Esquire” magazine from 2008 to 2012 and found that tall strong male models grace most of the covers. Furthermore, social culture often delivers the information that taller height can bring many benefits. Numerous studies have found that taller man have more advantages in hunting job (Judge and Cable, 2004; Watts, 2004; Gao and Smyth, 2012) and dating (Sheppard and Strathman, 1989; Nettle, 2002; Pawlowski and Koziel, 2002; Prokop and Fedor, 2011). A study of China’s urban labor survey across 10 years (2001–2010) showed that, even if at the same position, short workers receive less in wages than tall workers (Gao and Smyth, 2012). Huang (2008, unpublished) found that people are more likely to associate positive personality traits with taller individuals and negative personality traits with shorter individuals in a sample of Chinese college students. So social culture encourages individuals to pursue height ideals and engage in height comparisons, which in turn contribute to stature concerns.

However, few researchers have examined how appearance pressure and comparisons impact stature concerns. Only one known study has demonstrated that physical stature concerns were predicted by social pressure and comparisons independent of reported height and fatness concerns in a sample of Chinese male adolescents and young adults (Jackson and Chen, 2008b). But there is no study about female stature concerns, which is surprising since Chinese young women and men have reported the same concerns with stature (Chen et al., 2006) and women tend to be more susceptible to the influence of the social culture (Chen and Jackson, 2012; Luo, 2012). Therefore, we speculate that sociocultural factors can explain the height dissatisfaction of Chinese college students.

Objectification theory may also explain the height dissatisfaction. According to objectification theory (Fredrickson and Roberts, 1997), Western societies often sexually objectify the female body. That is, women’s bodies are objects to be looked at and evaluated. Such sexual objectification cause girls and women to internalize an observer’s perspective on their own bodies, a process referred to as “self-objectification,” thus treating themselves as an object to be looked at and evaluated on the basis of appearance. This adoption of the observer’s perspective of one’s own body is known as objectified body consciousness (OBC), which consists of three components: body surveillance, body shame, and appearance-control beliefs (McKinley and Hyde, 1996). Extensive research has indicated that features of OBC (body surveillance, body shame) were associated with body image concerns and disordered eating of adolescent girls and young women in the United States (e.g., Szymanski and Henning, 2007) and Australia (e.g., Tiggemann and Kuring, 2004). Moreover, studies on OBC have extended to other groups, such as young men in Australia (e.g., Tiggemann and Kuring, 2004), young women in South Korea (Kim et al., 2014) and young women and men in China (e.g., Jackson and Chen, 2015a,b). Recent studies have shown that body surveillance and body shame were associated with eating disorders (Jackson and Chen, 2015a), body dissatisfaction (Jackson et al., 2015), and cosmetic attitude (Jackson and Chen, 2015b) among young women and men in China.

To date, however, there is no research examining whether objectification theory extends to height dissatisfaction aside from weight/body dissatisfaction. According to objectification theory and OBC, body surveillance focuses on monitoring the body size and shape (Buchanan et al., 2008); however, it can be expanded to include other aspects of appearance. For example, Buchanan et al. (2008) found that habitual body monitoring of skin tone, rather than original body shape/size surveillance, predicted specific skin-tone dissatisfaction of African American women. Height is a salient physical attribute for Chinese adolescents and young adults, in addition to body size and shape (Chen et al., 2006). Chinese young women and men may expect to have their appearance evaluated not only in terms of body shape and size but also in terms of height. In keeping with the habitual skin-tone monitoring described by Buchanan et al. (2008) and face size and shape surveillance described by Kim et al. (2014), we hypothesized that Chinese young women and men similarly engage in habitual monitoring of their height, in addition to body shape/size surveillance.

This study therefore proposed to search for predictors of height dissatisfaction among college students in China from the perspective of social culture and objectification theory. Two hypotheses were tested. First, given that women are more susceptible to sexual objectification and sociocultural influences on appearance, women were expected to score significantly higher than men on measures of sociocultural model features (appearance pressure from mass media and close interpersonal networks, appearance social comparisons), OBC (body surveillance, body shame, stature surveillance), and stature dissatisfaction. Second, sociocultural model features and OBC were expected to explain significant, unique variance in stature concerns in each gender.

## Materials and Methods

### Participants and Procedures

The sample was comprised of 473 women and 231 men from a large university in Henan, China. Participants ranged from 16 to 22 years (*M* = 18.46, *SD* = 0.85) and 92.3% were of Han ethnicity. The BMI ranged from 15.57 to 29.32 (*M* = 20.47, *SD* = 2.45). The majority (68.3%) of the sample was of normal weight; 5.54% (*n* = 39) had a BMI of 25 or higher, the typical cut-off for defining overweight.

Data were collected via a web-based survey hosted by Wenjuan xing (a Chinese survey website). Participants were recruited from various optional psychology courses at a university in Henan, China. Participants were required to complete a questionnaire which comprised the measures of stature dissatisfaction, OBC (body surveillance, body shame, stature surveillance), sociocultural model features (i.e., appearance pressure from mass media and close interpersonal networks, appearance social comparisons), and demographic information (i.e., age, gender, height, and weight). This study was approved by the Ethics Committee of Henan University of Economics and Law. All participants submitted online informed consent before filling in the questionnaire. Participants received extra credit for their participation.

### Measurements

#### Negative Physical Self-Stature Concerns Subscale

The 11-item NPS-Stature (Chen et al., 2006) assesses thoughts, feelings, projections, and behaviors related to dissatisfaction with height (e.g., “I have been paying a lot of attention to my height”). Participants responded to each item on a five-point Likert scale ranging from 1 (never like me) to 5 (always like me). Sound reliabilities, stability, and validity have been reported in Chinese adolescent and young adult samples (Chen et al., 2006; Chen and Jackson, 2007). In this study, alphas were α = 0.92 for men and α = 0.93 for women.

#### Body Surveillance

Body surveillance was measured by the body surveillance subscale of the OBC scale (McKinley and Hyde, 1996). This subscale contains eight items that assess the frequency that participants monitor their physical appearance (e.g., “During the day, I think about how I look many times”). Participants responded to each item on a seven-point Likert scale ranging from 1 (strongly disagree) to 7 (strongly agree). The scale has satisfactory reliability and validity in Chinese samples (e.g., Jackson and Chen, 2015a,b). In this study, alphas were α = 0.79 for men and α = 0.78 for women.

#### Body Shame

Body shame was measured by the body shame subscale of the OBC scale (McKinley and Hyde, 1996); this subscale contains eight items that reflect the degree to which individuals feel shame about their bodies when they perceive themselves as not meeting the cultural standards of appearance (e.g., “I feel like I must be a bad person when I don’t look as good as I could”). Participants were required to respond on a seven-point Likert scale ranging from 1 (strongly disagree) to 7 (strongly agree). The scale has satisfactory reliability and validity in Chinese samples (Jackson and Chen, 2015a,b). In this study, alphas were α = 0.77 for men and α = 0.80 for women.

#### Stature Surveillance

Following Buchanan et al. (2008) and Kim et al. (2014), we created eight additional items to assess the culturally specific form of body monitoring regarding stature for Chinese sample: (a) “I often worry about how my height looks to other people”; (b) “I often compare my height with that of other people”; (c) “I rarely think about how my height looks”; (d) “I often think about how much taller or shorter my height is than other people’s”; (e) “I often wonder whether or not my height is attractive to other people”; (f) “I often think about how my height affects my looks”; (g) “I often feel conscious of how my height looks to other people”; and (h) “I often worry that my height is unattractive to other people.” Items are rated on a seven-point Likert-type scale (1 = strongly disagree to 7 = strongly agree). Higher scores indicate greater surveillance on height. PCA in this sample resulted in interpretable solutions (men: KMO: 0.904, Bartlett’s test: *p* < 0.001; women: KMO: 0.914, Bartlett’s test: *p* < 0.001). The eight items are loaded onto one factor (men: eigenvalue = 4.93; women: eigenvalue = 4.86), and explain 61.68 and 60.73% of the variance in stature surveillance among men and women, respectively. Stature surveillance scores correlated positively with body surveillance (men: *r* = 0.41, *p* < 0.001; women: *r* = 0.39, *p* < 0.001), and body shame (men: *r* = 0.37, *p* < 0.001; women: *r* = 0.43, *p* < 0.001). In this study, stature surveillance scores were internally consistent (men: α = 0.90; women: α = 0.90) and stable over a 5-week period (men: *r* = 0.87, *n* = 185; women: *r* = 0.88, *n* = 312). A multiple linear regression analysis was conducted using the Negative Physical Self-Stature Concerns subscale. The items of stature surveillance scale were the predictor variables. Results showed that all VIF values were below the commonly suggested cut-off of 10 (VIF < 3.6), indicating that multicollinearity was not a problem in this measure.

#### Sociocultural Attitudes Toward Appearance Questionnaire (SATAQ)–3–Pressure Subscale

Perceived mass-media pressure was assessed via six of seven SATAQ items (SATAQ-3-Pressure; Thompson et al., 2004). This subscale examined perceived pressure from television, movies, and magazines to conform to media portrayals of physical attractiveness. Items are rated on a five-point Likert scale ranging from 1 (definitely disagree) to 5 (definitely agree). Although the original four factor SATAQ-3 solution was not fully replicated in Chinese samples (Jackson and Chen, 2010), gender-equivalent single component solutions, including six of the seven original items and satisfactory psychometrics, were found in China-based studies using only Media Pressure items (Chen and Jackson, 2012). In this study, alphas were α = 0.88 for men and α = 0.90 for women.

#### Perceived Sociocultural Pressure Scale-Interpersonal (PSPS; Stice, 2001; Jackson and Chen, 2010)

Six Perceived Sociocultural Pressure Scale-Interpersonal (PSPS) items (Stice, 2001; Jackson and Chen, 2010) assessed perceived appearance pressure from friends, family, and dating partner (or best friend). Items were rated on a five-point Likert scale ranging from 1 (not at all) to 5 (very much). In Chinese samples, all six interpersonal PSPS items loaded on one component in Chinese samples of each gender (Chen and Jackson, 2012) and significant relations with body dissatisfaction and disordered eating (Jackson and Chen, 2010). In this study, alphas were α = 0.86 for men and α = 0.84 for women.

#### Physical Appearance Comparison Scale (PACS; Shroff and Thompson, 2006)

Four Physical Appearance Comparison Scale (PACS) items (Shroff and Thompson, 2006) assessed physical appearance comparison tendencies with others. Items were rated on a five-point Likert scale ranging from 1 (never) to 5 (always). The scale has good internal reliability and significant relations with body dissatisfaction and disordered eating in Chinese samples (Jackson and Chen, 2008a,b). In this study, alphas were α = 0.84 for men and α = 0.83 for women.

#### Demographics

Participants were asked to provide their age, gender, weight, height, and ethnicity. Body mass index (BMI) was calculated on the basis of self-reported height and weight (kg/m^2^).

## Results

**Table 1** displays the means and standard deviation for all variables separately for women and men. Since BMI and age significantly differed across gender, *F*(1,702) = 55.83, *p* < 0.01, $\eta_{p}^{2}$ = 0.07, *F*(1,702) = 7.39, *p* < 0.05, $\eta_{p}^{2}$ = 0.01, BMI and age were controlled for in analyses reported below. A multivariate analysis of variance with BMI and age as covariate was performed and a significant gender effect was revealed, *F*(10,691) = 218.67, *p* < 0.001, $\eta_{p}^{2}$ = 0.76. Univariate analyses of covariance on individual scales partially supported hypothesized gender differences. Consistent with our predictions, women had significantly higher scores than men on all measures of perceived appearance pressure from mass media and interpersonal sources, appearance comparisons with others, body surveillance, and body shame (see **Table 1**).

**Table 1:** Descriptive and univariate statistics for the main research measures among young Chinese women and men (*N* = 704).

	Sample				
	Men	Women		
	*M* ± *SD*	*M* ± *SD*	*F*	η
Stature concerns	22.69 ± 9.00	24.07 ± 9.79	4.32*	0.01
Height	175.88 ± 4.95	163.05 ± 4.82	1018.38**	0.59
Weight	66.52 ± 10.03	53.20 ± 6.42	999.95**	0.59
Body mass index	21.47 ± 2.95	20.00 ± 2.17	42.41**	0.40
Mass media pressure	12.83 ± 4.73	14.05 ± 4.57	90.18**	0.11
Interpersonal pressure	13.35 ± 4.87	14.05 ± 4.58	11.29**	0.02
Appearance comparisons	9.09 ± 3.36	10.51 ± 3.16	28.63**	0.04
Body surveillance	28.90 ± 8.51	32.17 ± 7.87	22.24**	0.03
Body shame	19.74 ± 7.32	20.39 ± 7.55	4.95*	0.01
Stature surveillance	24.03 ± 10.64	25.63 ± 10.67	4.28*	0.01

^∗^*p < 0.05; ^∗∗^*p* < 0.01*.

### Predictors of Stature Concerns among Men

Bivariate correlations indicated that stature concerns were negatively related to height and weight, but not to age and BIM. All sociocultural measures (appearance pressure from mass media, interpersonal appearance pressure, appearance social comparisons), and features of OBC (body surveillance, body shame, stature surveillance) had significant positive correlations with stature concerns (**Table 2**).

**Table 2:** Correlations between all variables for women and men.

Measure	1	2	3	4	5	6	7	8	9	10
(1) Stature concerns		-0.70**	-0.28**	0.05	0.20*	0.15*	0.17*	0.10*	0.19**	0.53**
(2) Height	-0.55**		0.40**	-0.06	0.33	0.03	0.07	0.06	0.06	-0.19*
(3) Weight	-0.17**	0.39**		0.89**	0.20**	0.20	0.25**	0.05	0.21*	-0.05
(4) Body mass index	0.03	0.02	0.92**		0.21**	0.24**	0.08	0.02	0.20**	-0.05
(5) Mass media pressure	0.42**	-0.03	0.10	0.12		0.72**	0.67**	0.37**	0.51**	0.35**
(6) Interpersonal pressure	0.32**	0.05	0.16**	0.16**	0.75**		0.74**	0.42**	0.50**	0.35**
(7) Appearance comparisons	0.34**	0.01	0.06	0.07	0.76**	0.82**		0.48**	0.49**	0.44**
(8) Body surveillance	0.17**	0.05	-0.03	0.07	0.34**	0.44**	0.56**		0.31**	0.39**
(9) Body shame	0.22**	0.07	0.08	0.06	0.47**	0.48**	0.40**	0.30**		0.43**
(10) Stature surveillance	0.53**	0.11	-0.03	0.01	0.56**	0.52**	0.55**	0.41**	0.37**	

^∗^*p < 0.05; ^∗∗^*p* < 0.01. Correlations for women (*n* = 473) in upper diagonal; correlations for men (*n* = 231) in lower diagonal*.

Hierarchical multiple regression analyses were conducted to examine the relationship between sociocultural variables and features of OBC in stature concerns. To evaluate and control for the impact of relevant background influences, significant demographics were entered in Block 1 of the prediction models. To test the hypothesis that sociocultural factors would influence stature concerns, features of sociocultural model (appearance pressure from mass media, interpersonal appearance pressure, appearance social comparisons) were entered in Block 2, independent of demographic correlates. Body surveillance, body shame, and stature surveillance were entered in Block 3 to assess how features of OBC would contribute to prediction models independent of significant measures of sociocultural factors and demographic correlates.

**Table 3** presents the results of the final prediction model for men. In the prediction model for stature concerns, Block 1 demographics, height, and weight explained substantial variance in the criterion (Adj. *R*^2^ = 0.31). After controlling for the impact of Block 1 demographics, measures of sociocultual model features (Block 2) predicted significant additional variance (Adj. *R*^2^ = 0.17) in stature concern levels in men; only mass media appearance pressure had a unique impact within Block 2. Higher levels of reported mass media appearance pressure had significant unique associations with elevations in stature concerns. Finally, consistent with our predictions, after controlling for the impact of sociocultural model measures and demographics, features of OBC combined with significant additional variance in stature concerns, Adj. *R*^2^ = 0.09; stature surveillance had the strongest unique impact within Block 3. Overall, the eight predictors explained Adj. *R*^2^ = 0.56 in stature concerns of men.

**Table 3:** Predictors of stature concerns among young adult Chinese women and men.

		Men (*n* = 231)				Women (*n* = 473)			
Block	*Predictors*	β	95%CI	*T*	f^2^	β	95%CI	*T*	f^2^
1	Height	-0.57	[-1.25, -0.82]	-9.51^∗∗∗^		-0.70	[-1.57, -1.28]	-19.67^∗∗∗^	
	Weight	0.05	[-0.06, 0.15]	0.84		0.01	[-0.09, 0.11]	0.11	
		
		*R*^2^ Ch. = 0.31, *F*(2,228) = 50.16^∗∗∗^			0.96	*R*^2^ Ch. = 0.49, *F*(2,470) = 229.01^∗∗∗^			0.43
		
2	Height	-0.54	[-1.18, -0.78]	-10.27^∗∗∗^		-0.70	[-1.56, -1.29]	-20.68^∗∗∗^	
	Weight	-0.01	[-0.10, -0.09]	-0.13		-0.04	[-0.16, 0.04]	-1.19	
	Mass media pressure	0.34	[0.34, 0.91]	4.28^∗∗∗^		0.13	[0.09, 0.48]	2.84^∗∗^	
	Interpersonal pressure	0.06	[-0.21, 0.42]	0.68		0.02	[-0.16, 0.23]	0.38	
	Appearance comparisons	0.03	[-0.40, 0.57]	0.36		0.13	[0.10, -0.70]	2.58^∗^	
		
		*R*^2^ Ch. = 0.47, *F*(5,225) = 40.37^∗∗∗^			1.23	*R*^2^ Ch. = 0.56, *F*(5,467) = 117.18^∗∗∗^			0.86
		
3	Height	-0.51	[-1.11, -0.75]	-10.38^∗∗∗^		-0.61	[-1.38, -1.13]	-20.25^∗∗∗^	
	Weight	0.01	[-0.08, 0.10]	0.27		-0.04	[-0.14, 0.03]	-0.13	
	Mass media pressure	0.22	[0.12, 0.68]	2.81^∗∗^		0.09	[0.01, 0.36]	2.09^∗^	
	Interpersonal pressure	0.01	[-0.28, 0.32]	0.12		0.02	[-0.14, 0.20]	0.37	
	Appearance comparisons	-0.04	[-0.60, 0.40]	-0.40		0.01	[-0.25, 0.31]	0.22	
	Body surveillance	-0.01	[-0.13, 0.11]	-0.22		-0.07	[-0.16, -0.01]	-2.14^∗^	
	Body shame	0.02	[-0.10, 0.16]	0.43		0.04	[-0.03, 0.14]	1.31	
	Stature surveillance	0.37	[0.22, 0.41]	6.42^∗∗∗^		0.38	[0.29, 0.41]	11.40^∗∗∗^	
		
		*R*^2^ Ch. = 0.56, *F*(8,222) = 35.31^∗∗∗^			1.94	*R*^2^ Ch. = 0.67, *F*(8,464) = 116.37^∗∗∗^			1.19

^∗^*p < 0.05; ^∗∗^*p* < 0.01; ^∗∗∗^*p* < 0.001*.

### Predictors of Stature Concerns among Women

For women stature concerns were negatively related to height and weight (**Table 2**, lower diagonal), which were entered in Block 1 of the prediction model. All sociocultural measures (appearance pressure from mass media, interpersonal appearance pressure, appearance social comparisons), body surveillance, body shame, and stature surveillance had significant positive correlations with stature concerns.

**Table 3** presents the results of the final prediction model for women. In the prediction model for stature concerns, after controlling for the impact of height and weight (Block 1, Adj. *R*^2^ = 0.49) measures of sociocultural model features (Block 2) predicted significant additional variance (Adj. *R*^2^ = 0.06) in stature concerns levels of women; mass media appearance pressure and appearance social comparisons had unique effects on stature concerns of women. However, appearance comparisons did not remain a significant predictor when the features of OBC were entered in the final step. Also as hypothesized, features of OBC combined with significant additional variance in this criterion (Adj. *R*^2^ = 0.09), beyond the impact of all other predictors; stature surveillance had the strongest unique impact within this block, although body surveillance made a modest unique contribution (Block 3). Overall, the eight predictors explained Adj. *R*^2^ = 0.67 in stature concerns of women.

## Discussion

Stature concerns are a prominent source of body dissatisfaction for Chinese teenagers and young adults (Chen et al., 2006; Jackson and Chen, 2008b). However, little is known about the psychological factors that account for this body concern among young women and men in China. This study combined social cultural model and objectification theory to explore the predictors of this culturally salient appearance concern.

Among Chinese young men, height was a robust predictor of stature concerns. This result was consistent with previous studies (Jackson and Chen, 2008b), Chinese young men who reported shorter height experienced greater dissatisfaction about height. This association may due to taller stature as a salient facet of masculine attractiveness ideals for Chinese young men (Chen et al., 2006), so shorter men may recognize discrepancies from taller body ideals and engender negative reactions for deviating from this standard.

Appearance pressure from the mass media was a robust predictor of stature concerns among Chinese young men. This is in line with other studies of younger Chinese males (Jackson and Chen, 2008b), men who perceived more pressure from the mass media were more likely to be dissatisfied with their height. In fact, past research has found that appearance pressure from the mass media is an important source of body image concern (e.g., Chen et al., 2006; Chen and Jackson, 2008). Because the ideal body size portrayed by the mass media is difficult to achieve, individuals become dissatisfied with their bodies. Male characters in the media are usually taller, since the ideal of a beautiful male body includes not only strong muscles, but also a tall figure (Hua, 2013, unpublished). The mass media also often conveys the message that “taller is better.” In China the mass media calls a male ideal date “gao fu shuai.” Gao means taller stature, fu means wealth, shuai means handsome facial appearance. Height is listed first, showing that it is considered more important than wealth and beauty. Thus, this appearance pressure from mass media can contribute to stature concerns of male college students in China.

As hypothesized, stature surveillance explained significant unique variance in stature concerns among Chinese men. The more frequently they monitor their height, the more likely they are to perceive a defect and be dissatisfied. As is common knowledge, objectification theory was originally proposed to examine how women’s socialization and experiences of sexual objectification are translated into mental health problems (Fredrickson and Roberts, 1997). Women who live in sexually objectifying environments, are more likely to objectify themselves (Vandenbosch and Eggermont, 2012), experience more body shame and appearance anxiety (e.g., Tiggemann and Kuring, 2004), and suffer from eating disorders (e.g., Calogero, 2009), depression (e.g., Szymanski and Henning, 2007), sexual dysfunctions (e.g., Steer and Tiggemann, 2008), body dissatisfaction (e.g., Jackson et al., 2015), and impaired cognitive performance (Gay and Castano, 2010). However, there is some evidence to suggest that men are increasingly sexually objectified as well (e.g., Rohlinger, 2002), and similarly suffer the theorized consequences of sexual objectification (Calogero, 2009). For example, Hebl et al. (2004) found that like women, men had higher body shame and lower self-esteem when put in a self-objectifying situation. Some studies have shown that body surveillance and self-objectification can predict eating disorders and body image concerns of young men in Australia (e.g., Tiggemann and Kuring, 2004), the United States (e.g., Wiseman and Moradi, 2010), and China (e.g., Jackson et al., 2015). As with women, men may internalize an observer’s perspective on their own bodies, causing them to show excessive attention to their own bodies and evaluate their self-worth on the basis of appearance. However, this proposed body surveillance by objectification theory focused on monitoring body shape or size, rather than height (Buchanan et al., 2008). Given the ideal body image is multidimensional among young adults in China (Chen et al., 2006), we posit that body monitoring includes not only general body shape monitoring, but also height monitoring. Consistent with our hypothesis, our results showed that stature surveillance as unique variance in stature concerns, but body surveillance and shame did not. This suggests that objectification theory can also be used to explain stature concerns among young male in china.

Among young Chinese women, correlates and unique predictors of stature concerns were very similar to those of men. The appearance pressure from the media and stature surveillance explained significant unique variance in stature concerns independent of height. Women who perceive more pressure from the mass media and frequently monitor their height are also more likely to be dissatisfied with their height. However, unlike men, women’s general body surveillance can also predict stature concerns. This result is consistent with the research on the Chinese physical ideal. Chen et al. (2006) found that for the characteristics of the Chinese adolescent ideal body, men focus on taller stature and strength while women emphasize symmetry and thinness. This suggests that young men and women have different perceptions of the ideal body type under the cultural background of China. For Chinese young women, they are more concerned about whether their weight is appropriate for their height. Thus, their general body size surveillance can contribute to stature concerns.

A noteworthy finding is that appearance pressure from mass contributes substantially more to stature concerns in males than females. This pattern suggests that height dissatisfaction for young females in China is less strongly related to appearance pressure from mass than it is for males. This is surprising, as past research has demonstrated that compared to Chinese men, Chinese women are more vulnerable to environmental pressures that result in dissatisfaction with their bodies (e.g., Chen et al., 2007). Consistent with this, our results indicated that Chinese women have significantly higher scores than men on all variables. One possible explanation for this finding is that mass media in China often gives young men the message that a taller stature garners greater advantages in life (Watts, 2004; Gao and Smyth, 2012). Such social pressure may contribute more to stature concerns in males.

Taken together, our findings demonstrate that appearance pressure from the mass media and stature surveillance are robust predictors of stature concerns for both genders independent of height. More specifically, the pressures from height ideals and the information that “taller is better” portrayed by the mass media contribute to stature concerns for young men and women in China. Thus, some prevention and intervention programs that educated young men and women to resist these pressures may have potentially far-reaching benefits to reduce stature concerns. The present findings also suggest that stature is a relevant dimension of habitual body monitoring for Chinese young men and women, and that this constant body monitoring plays a significant role in promoting stature dissatisfaction. This finding suggests that it is necessary and important to consider culture specific aesthetic standards with diverse groups experiencing specific culturally salient appearance concerns.

Like all research, the findings of the present study should be interpreted in light of a number of limitations. First, because the sample consisted of Chinese university students, the results may not be generalizable to other samples. Future research should include larger and more diverse samples. Second, similar to previous research, our data were collected at a single time point (Chen and Jackson, 2008). Thus, no causal claims can be made about the relations between the variables. Future research should implement studies with longitudinal designs to explore the development of these constructs, as well as investigate potential causal relations between these variables across time. Thirdly, the entirety of the framework of objectification theory was not applied in this study. We only evaluated the impact of key objectification theory constructs (body surveillance and body shame) on stature concerns. Future research should consider more features of objectification theory in the prediction model for stature concerns. Finally, although sociocultural factors and features of OBC explained the significant unique variance in stature concerns among young men and women in China, other factors may also influence stature concerns; these can include stereotypes (Cameron et al., 1977), stigma (Martel and Billier, 1987), and sexist beliefs (Swami et al., 2010). Future research should consider more factors in the prediction model for stature concerns.

## Conclusion

This study may be the first to assess the impact of sociocultural factors and features of OBC on stature concerns among young Chinese women and men. Results indicated that for Chinese male and female university students, appearance pressure from the media and stature surveillance are reliable predictors of individual stature concerns, independent of height. These results extend the sociocultural models and objectification theory to some specific body image concerns, which has the cross-cultural significance.

## Author Contribution

QS carried out the experimental work and the data collection, interpretation, and wrote the manuscript.

## Conflict of Interest Statement

The author declares that the research was conducted in the absence of any commercial or financial relationships that could be construed as a potential conflict of interest. The reviewer AB and handling Editor declared their shared affiliation, and the handling Editor states that the process nevertheless met the standards of a fair and objective review.
